# Cost-Effectiveness of Canagliflozin Versus Dapagliflozin Added to Metformin in Patients With Type 2 Diabetes in China

**DOI:** 10.3389/fphar.2019.00480

**Published:** 2019-05-08

**Authors:** Xingyun Hou, Xu Wan, Bin Wu

**Affiliations:** ^1^Department of Pharmacy, Changzheng Hospital, Second Military Medical University, Shanghai, China; ^2^Medical Decision and Economic Group, Department of Pharmacy, Ren Ji Hospital, South Campus, School of Medicine, Shanghai Jiao Tong University, Shanghai, China

**Keywords:** canagliflozin, dapagliflozin, cost-effectiveness, type 2 diabetes mellitus, incremental cost-effectiveness ratio

## Abstract

**Purpose:**

Agents that inhibit sodium glucose co-transporter 2 (SGLT2), including canagliflozin and dapagliflozin, become available for the treatment of Chinese patients with type 2 diabetes mellitus (T2DM). This study assessed the economic outcomes of canagliflozin 100 mg versus dapagliflozin 10 mg in patients with T2DM inadequately controlled with metformin in the Chinese context.

**Materials and Methods:**

Economic outcomes were projected by using the validated Chinese Outcomes Model for T2DM (COMT). Efficacy and safety, medical expenditure, and utility data were derived from the literature, which were assigned to model variables for estimating the quality-adjusted life-years (QALYs) and costs as well as incremental cost-effectiveness ratios (ICERs). The analysis was conducted from the perspective of Chinese healthcare service providers. One-way and probabilistic sensitivity analyses were performed. Health outcomes and costs were discounted at 5%.

**Results:**

Relative to dapagliflozin 10 mg, treatment with canagliflozin 100 mg was associated with additional 0.015 expected life years per patients treated and 0.013 QALYs gained, which was driven by the reduced risk of macrovascular and microvascular complications over lifetime horizon. The incremental cost of canagliflozin 100 mg versus dapagliflozin 10 mg was US $-129, which indicated the canagliflozin 100 mg strategy was a dominant option. The univariate sensitivity analyses indicated that the results were sensitive to several model inputs.

**Conclusion:**

These results suggested that canagliflozin was a cost-saving treatment option compared with dapagliflozin from the perspective of Chinese health care services providers for Chinese patients with T2DM who are inadequately controlled on metformin monotherapy.

## Introduction

The recent Global Burden of Disease Study showed that all-age disability-adjusted life-years (DALYs) for diabetes mellitus in 2017 were 35,000 (95% confidence interval [CI]: 28,500–42,700) thousands, of which type 2 diabetes mellitus (T2DM) accounted for about 85% (GBD 2017 DALYs and HALE Collaborators, 2018). One recent Chinese report also indicated that China has a huge disease burden related to diabetes: about 25% of people with diabetes worldwide are Chinese, where nearly one in eleven people has diabetes and one half of them has prediabetes (Xu et al., 2013; Wang et al., 2017). The financial burden caused by diabetes in Chinese economic augmented from 2.216 billion Chinese yuan in 1993 to 200 billion Chinese yuan in 2007 (Chan et al., 2014; Wang H. et al., 2015). Based on Chinese guidelines (Chinese Diabetes Society, 2014), metformin is used as the first-line drug for controlling hyperglycemia to decrease the risk of complications related to T2DM. However, with the passage of time it may not adequately controlling hyperglycemia due to disease progression which necessitates add-on treatments to keep euglycemia, such as DPP-4 inhibitors and GLP-1 receptor agonists.

By stimulating glucose excretion in the urine, the sodium glucose co-transporter 2 (SGLT2) inhibitors have been approved as the most recent class of therapies for managing T2DM (Li et al., 2017). In several clinical trials with T2DM, SGLT2 inhibitors, such as canagliflozin, empagliflozin and dapagliflozin, are found to decrease HbA1c, fasting plasma glucose (FPG) levels, and body weight by inducing favorable glucosuria (urinary loss of approximately 200–300 kcal/d) in a variety of designs involving monotherapies and combination therapies (Storgaard et al., 2016). In China, canagliflozin and dapagliflozin has become available. Based on the recent studies, canagliflozin 100 mg showed enough efficacy to achieve glycemic control and was well tolerated in adults of Eastern Asian (Inagaki et al., 2018). According to indirect comparisons with Bayesian network meta-analysis, the favorable efficacy was achieved with canagliflozin 100 mg versus dapagliflozin 10 mg (Zaccardi et al., 2016). As a chronic and progressive disease, the financial burden of T2DM over the long run is considerable. Efficient use of health resources is necessary in order to inform the decision-making makers. The economic outcomes of SGLT2 inhibitors versus other antidiabetic agents have been reported (Johnston et al., 2017). However, there have been only a paucity of reports of head-to-head cost-effectiveness evaluations of SGLT2 inhibitors (Neslusan et al., 2018). By using our recently reported Chinese Outcomes Model for T2DM (COMT; Li T. et al., 2018; Wu et al., 2018a), the objective of this analysis is to provide cost-effectiveness evidence regarding the use of canagliflozin 100 mg and dapagliflozin 10 mg for treating adult patients with T2DM uncontrolled on background metformin therapy.

## Materials and Methods

### Model Overview

**FIGURE 1:**
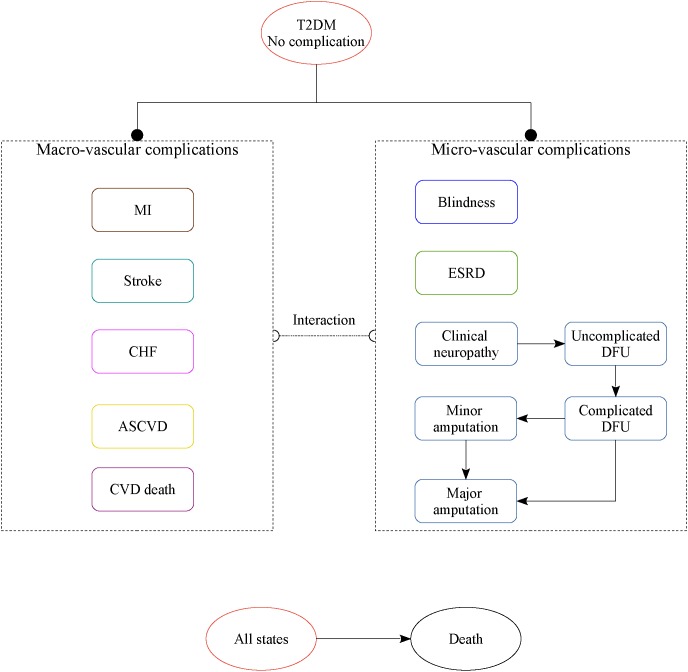
The structure of the Chinese T2DM health policy model.

This study provides an economic analysis of SGLT2 inhibitors treatment for T2DM patients with inadequate glycemic control on metformin monotherapy who were initially assigned to canagliflozin 100 mg or dapagliflozin 10 mg strategy. The analysis was carried out using the COMT (Li T. et al., 2018; Wu et al., 2018a), a validated Chinese diabetes policy analysis model that would track several key diabetic macro- and micro-vascular complications for one hypothetical T2DM patient (Figure 1), including myocardial infarction (MI), congestive heart failure (CHF), cardiovascular disease (CVD), stroke, blindness, end-stage renal disease (ESRD), clinical neuropathy, foot ulcer, minor and major amputation. The all-cause mortality would be adjusted based on the treatment effect and disease status. Each diabetic complication is an independent sub-model that was integrated with the COMT model. The transition probabilities of the model were collected from the published reports. During the model simulation, interconnectivity and interaction among sub-models of individual complication was permitted to allow the complication risks to be updated by using tracker parameters. The clinical and demographic characteristics of the hypothetical cohorts with T2DM were used for determining the annual disease progression: sex, age, smoking status, systolic blood pressure (SBP), glycated hemoglobin (HbA1c), total and high-density lipoprotein (HDL) cholesterol levels, serum creatinine, urine albumin:creatinine ratio, history of cardiovascular disease, use of antihypertensive, anticoagulant medications, statin and oral diabetes medication. During the simulation, risk parameters might be adjusted based on the treatment transition, thereby resulting in the likelihood of complication incidence. More details of the model could be found in our previous work (Wu et al., 2018a).

To keep with other economic reports associated with T2DM therapies (Charokopou et al., 2016), the current analysis used the lifetime health and economic outcomes as the main endpoints, including costs, cumulative probabilities of diabetic complications, life year (LY), quality-adjusted life year (QALY) and incremental cost-effectiveness ratios (ICERs). The annual costs and QALY were discounted at 5% according to the Chinese health economics recommendations (Wang X. et al., 2015). When the ICER of canagliflozin 100 mg against dapagliflozin 10 mg was lower than the per capita gross domestic product (GDP) of China in 2017 ($9,117) that was used as the willingness-to-pay threshold, canagliflozin 100 mg strategy would be deemed to be cost-effective (Wang X. et al., 2015). The current study was based on model techniques and literature review and did not require approval by the Institutional Research Ethics Board.

### Patient Profile and Treatment Effects

The patient characteristic profiles of receiving SGLT2 inhibitors were reported in a clinical trial that enrolled 444 Asian patients with D2DM after metformin failure (Yang et al., 2016). When data pertaining to a specific parameter that was used for estimating the complications (Basu et al., 2017), such as history of smoking and anticoagulation usage, was not available, information from Chinese national cross-sectional were used as a reference (Yang et al., 2010; Xu et al., 2013; Wang et al., 2017). In the literature review by searching PubMed and Web of Knowledge, we electronically searched randomized controlled trials (≥24 weeks) including canagliflozin and dapagliflozin that were published up to 1 November 2018. However, no head-to-head comparisons of canagliflozin 100 mg and dapagliflozin 10 mg was reached. Thus, the Clinical data was derived from a network meta-analysis, which synthesized the treatment efficacy and safety data reported by clinical trials (Zaccardi et al., 2016). In this indirect comparisons, placebo arm was used as the reference (Table 1). The mean absolute changes from baseline in HbA1c levels, SBP, total and HDL cholesterol were presented in Table 1, which were employed in the first year of therapy. In the subsequent year, HbA1c was mimicked to rise naturally (non-linear fashion), due to progressive nature of the disease, according to the HbA1c trajectories analysis. Similar assumptions were made for SBP, total and HDL cholesterol. The urinary tract infections and genital infections are the most common adverse effects for SGLT2 inhibitors (Li et al., 2017). The annual incidences of canagliflozin and dapagliflozin were estimated based on previous studies (Neal et al., 2017; Neslusan et al., 2018).

**Table 1:** Treatment effects comparing with placebo.

Parameters	Dapagliflozin 10 mg		Canagliflozin 100 mg		Source
	Expected value	Range	Expected value	Range	
HbA1c (%), decreasing	0.66	0.58 ~ 0.74	0.76	0.58 ~ 0.74	Zaccardi et al., 2016
SBP (mmHg), decreasing	3.01	2.13 ~ 3.89	3.89	2.88 ~ 4.9	Zaccardi et al., 2016
Total-C (mg/dL), decreasing	-4.72	-5.51 ~-3.92	4.56	4.5 ~ 4.62	Zaccardi et al., 2016
HDL-C (mg/dL), increasing	2.32	1.55 ~ 3.48	2.71	1.55 ~ 3.87	Zaccardi et al., 2016


*^∗^The reported data from trials were synthesized by using network meta-analysis.*

### Costs and Utilities

The current analysis was carried out from the perspective of Chinese health care services providers, and only direct medical costs related to T2DM and its complications were considered in this analysis (Table 2). All cost data are showed as 2017 US dollars (1 US $ = 6.8 Chinese Yuan). According to Chinese trials in adults of Eastern Asian (Inagaki et al., 2018), the daily dosage of canagliflozin and dapagliflozin were 100 and 10 mg per day. The prices of canagliflozin and dapagliflozin were derived from local hospitals. The costs of anti-diabetic therapy and glucose testing strips related to T2DM were collected from a large national population-based screening study that interviewed 1,482 adults with T2DM at 12 Chinese sites (Yang et al., 2012). Other potential consumption of health resources, such as the costs of outpatient visits and hospitalization associated with diabetic complications, which were extracted directly from published literature or other local sources, were also reflected in the simulation (Li et al., 2015; Wang H. et al., 2015; Gu et al., 2016; Shao et al., 2017; Wu et al., 2018b,c). The costs of adverse events, including hypoglycemic events, urinary tract and genital infections were derived from a Chinese cost studies (Ya-Ming et al., 2012; Shao et al., 2017).

**Table 2:** Key model inputs of costs and utilities.

Parameters	Expected value	Range	Source
*Costs ($)*			
Dapagliflozin 10 mg per day	2.51	1.25 ~ 2.51	Local charge
Canagliflozin 100 mg per day	2.46	1.23 ~ 2.46	Local charge
Anti-diabetic therapy per day (Disease duration ≤ 3 year)	0.5	0.2 ~ 1.3	Yang et al., 2012
Anti-diabetic therapy per day (3 < Disease duration ≤ 5 year)	0.8	0.2 ~ 1.7	Yang et al., 2012
Anti-diabetic therapy per day (5 < Disease duration < 10 year)	1.2	0.3 ~ 2.5	Yang et al., 2012
Anti-diabetic therapy per day (Disease duration ≥ 10 year)	2.0	0.7 ~ 3.2	Yang et al., 2012
MI hospitalization per event	7,383.0	6505.2 ~ 8260.9	Li et al., 2015; Wang H. et al., 2015; Gu et al., 2016; Shao et al., 2017
Care after MI per year	455.4	288.6 ~ 622.2	Li et al., 2015; Wang H. et al., 2015; Gu et al., 2016; Shao et al., 2017
Stroke hospitalization per event	2,875.2	2184.6 ~ 4738.3	Li et al., 2015; Wang H. et al., 2015; Gu et al., 2016; Shao et al., 2017
Care after stroke per year	506.9	445.9 ~ 828	Li et al., 2015; Wang H. et al., 2015; Gu et al., 2016; Shao et al., 2017
CHF per year	1,507.7	1254.6 ~ 2632.3	Gu et al., 2016; Li et al., 2015; Wang H. et al., 2015; Shao et al., 2017
ESRD per year	13,803.2	13153.8 ~ 14569.2	Wu et al., 2018c
Blindness per year	1,642.0	1430.4 ~ 1853.5	Li et al., 2015; Wang H. et al., 2015; Gu et al., 2016; Shao et al., 2017
Clinical neuropathy per month	60.9	26.2 ~ 101.4	Wu et al., 2018b
Uncomplicated DFU per event	76.2	0 ~ 226.2	Wu et al., 2018b
Complicated DFU per event	2,293.3	1228.5 ~ 2880.8	Wu et al., 2018b
Minor amputation per event	3,316.9	2165.2 ~ 5038.9	Wu et al., 2018b
Major amputation per event	5,019.2	2981.1 ~ 7738.2	Wu et al., 2018b
Care after major amputation per month	338.1	0 ~ 600.7	Wu et al., 2018b
Hypoglycemia per event	70.0	0 ~ 855.5	Local charge
Urinary tract infections per event	31.00	23.3 ~ 38.8	Shao et al., 2017
Genital infections per event	31.00	23.3 ~ 38.8	Shao et al., 2017
*Health utility scores*			
T2DM without complications	0.876	0.736 ~ 1	Pan et al., 2016
*Health disutility scores*			
Stroke hospitalization for 1 month	1.000	0.326 ~ 1	Pan et al., 2016
Stroke after discharge	0.326	0.036 ~ 0.616	Pan et al., 2016
MI hospitalization for 1 month	1.000	0.236 ~ 1	Pan et al., 2016
MI after discharge	0.236	0.026 ~ 0.446	Pan et al., 2016
CHF	0.236	0.026 ~ 0.446	Pan et al., 2016
ESRD	0.400	0.19 ~ 0.61	Li et al., 2015; Wang H. et al., 2015; Gu et al., 2016; Shao et al., 2017; Wu et al., 2018b,c
Blindness	0.157	0.007 ~ 0.307	Li et al., 2015; Wang H. et al., 2015; Gu et al., 2016; Shao et al., 2017; Wu et al., 2018b,c
Clinical neuropathy	0.185	0.015 ~ 0.355	Pan et al., 2016
Uncomplicated DFU	0.250	0.213 ~ 0.287	Li et al., 2015; Wang H. et al., 2015; Gu et al., 2016; Shao et al., 2017; Wu et al., 2018b,c
Complicated DFU	0.300	0.165 ~ 0.435	Li et al., 2015; Wang H. et al., 2015; Gu et al., 2016; Shao et al., 2017; Wu et al., 2018b,c
Minor amputation	0.320	0.204 ~ 0.436	Li et al., 2015; Wang H. et al., 2015; Gu et al., 2016; Shao et al., 2017; Wu et al., 2018b,c
Major amputation	0.380	0.264 ~ 0.496	Li et al., 2015; Wang H. et al., 2015; Gu et al., 2016; Shao et al., 2017; Wu et al., 2018b,c
Urinary tract infections per event	0.030	0.023 ~ 0.038	Shao et al., 2017
Genital infections per event	0.030	0.023 ~ 0.038	Shao et al., 2017


*MI, myocardial infarction; CHF, congestive heart failure; CVD, cardiovascular disease; ESRD, end-stage renal disease; DFU, diabetic foot ulcer.*

Health utility scores were collected from a recent reports that included 289 Chinese patients with T2DM and investigated the health state utility scores of diabetes without complications, neuropathy, heart and cerebrovascular disease by using the validated Chinese EQ-5D-5L instrument (Pan et al., 2016). Other utility scores that were not reported in this study, such as ESRD, minor and major amputation, were retrieved from published literatures (Li et al., 2015; Wang H. et al., 2015; Gu et al., 2016; Shao et al., 2017; Wu et al., 2018b,c). The disutility’s related to urinary tract and genital infections were 0.03 (Shao et al., 2017).

### Sensitivity Analyses

To test the robustness of model outputs, both univariate and probabilistic sensitivity analyses (PSA) were carried out. In univariate sensitivity analyses, the incremental net-health benefit (INHB) would be calculated based on the following formula: INHB(λ) = (μ_E1_ - μ_E0_) - (μ_C1_ - μ_C0_)/λ = Δ*E* - Δ*C*/λ, where μ_Ci_ and μ_Ei_ were the cost and effectiveness of treatment (*i* = 1) or control (*i* = 0), respectively, and λ was the willingness-to-pay threshold (Craig and Black, 2001). The values of model inputs were changed in the univariate sensitivity analysis, whose ranges were extracted from the published literatures (Table 1). When the relevant ranges were not reported, a range of 75–125% of the base-case values would be adopted. In the PSA, 1,000 iterations of second-order Monte Carlo simulations was conducted, where parameters were designated with probability distributions. Beta distribution were attached to the probability, proportions, utility and disutility scores; triangle distribution to cost estimates; and normal distribution to hazard ratio and patient characteristic profile. If standard error was not available, 25% of the reported base-case value would be applied. The results of the PSA were presented as a cost-effectiveness acceptability curve (CEAC).

## Results

### Base-Case Outcomes

In comparison with dapagliflozin 10 mg strategy, canagliflozin 100 mg strategy gained average health benefits in life expectancy and QALY of 0.015 years and 0.013 QALYs, respectively, at less total mean costs of $129 over a patient’s lifetime (Table 3), leading to an ICER of $-9,781 per QALY gained. These health augments in the canagliflozin 100 mg treatment arm were driven by the reduced cumulative incidence of macrovascular and microvascular complication.

**Table 3:** Base-case results for canagliflozin 100 mg strategy vs. dapagliflozin 10 mg strategy over a lifetime horizon.

	Dapagliflozin	Canagliflozin
Outcomes	10 mg	100 mg	Difference^∗^
**Cumulative probabilities**			
**of events**
MI (%)	9.33	9.29	-0.04
Stroke (%)	21.04	20.85	-0.19
CHF (%)	14.13	14.11	-0.02
ESRD (%)	3.709	3.702	-0.01
Blindness (%)	4.11	4.11	0.00
Clinical neuropathy (%)	14.64	14.63	-0.01
Minor amputation (%)	11.43	11.42	0.00
Major amputation (%)	8.43	8.43	0.00
Total QALY	10.32	10.34	0.013
Total LY	21.67	21.68	0.015
Total overall cost (US $)^#^	14,958	14,829	-129
ICER (US $/QALY)	NA	–9,781 (Dominant)^∗^	


*^∗^Compared with the Dapagliflozin 10 mg strategy. ^#^The total overall cost included the direct medical costs related to managing type 2 diabetes mellitus, such as the cost of medicines and managing complications.*

### Sensitivity Outcomes

**FIGURE 2:**
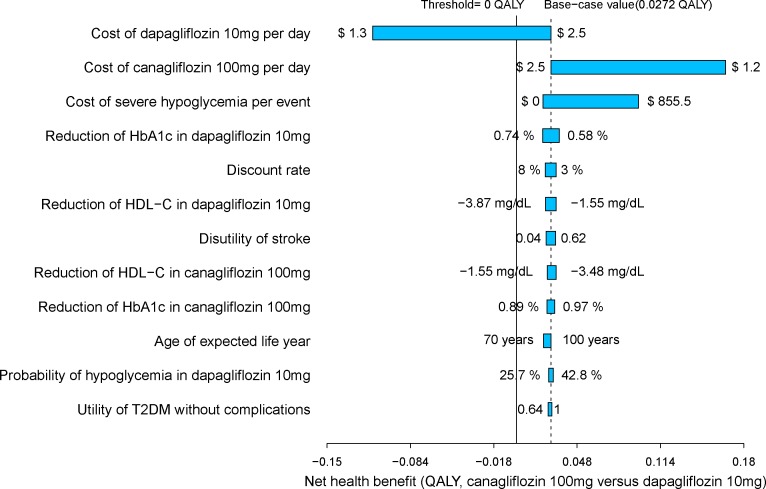
Tornado diagram for canagliflozin 100 mg strategy vs. dapagliflozin 10 mg strategy.

The univariate sensitivity analyses revealed that the results of the model were more sensitive to the cost of canagliflozin 100 mg and dapagliflozin 10 mg strategy because they were found to have a substantial impact on the economic outcomes. The remainder of the sensitive variables, such as the disutility values and the costs of complications, had a moderate or small impact (Figure 2).

**FIGURE 3:**
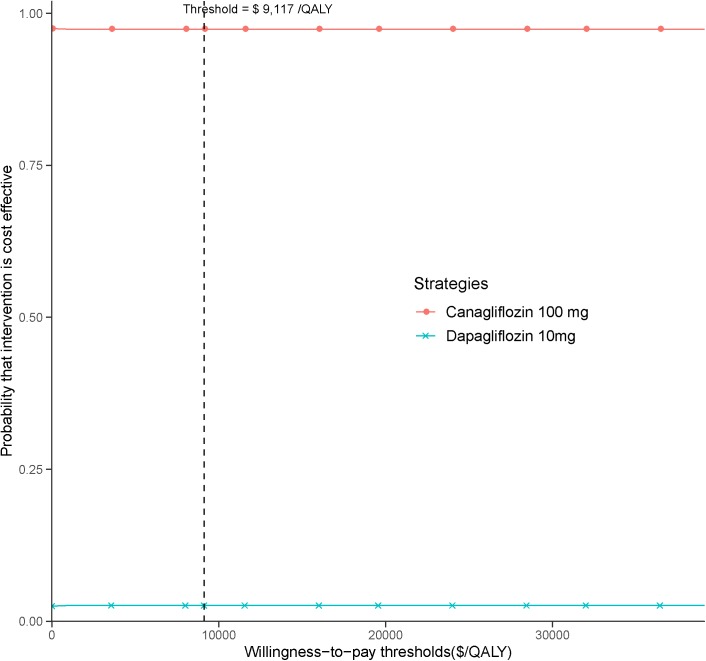
Cost-effectiveness acceptability curve for canagliflozin 100 mg strategy vs. dapagliflozin 10 mg strategy.

In the PSA, canagliflozin 100 mg strategy produced a nearly 95% probability of cost-effectiveness at an acceptable ICER of $9,117 (three times the GDP per capita of China in 2017), as shown in the CEAC (Figure 3).

## Discussion

Reports of the health benefits of SGLT2 inhibitors in clinical trials have caused great excitement among both decision makers and patients. However, the widespread use of SGLT2 inhibitors comes with a considerable increase in health resource consumption compared with other antidiabetic agents, which is of concern to patients and payers. The need for identifying the most cost-effective SGLT2 inhibitor is becoming urgent in the Chinese context. The current economic analysis indicates that canagliflozin 100 mg dominated dapagliflozin 10 mg as add-on to metformin from the perspective of Chinese health care services providers. More health benefits and lower costs were found with canagliflozin against dapagliflozin in the majority of simulations, suggesting a reasonable level of certainty. To the best of our knowledge, this is the first economic study examined the outcomes of the addition of canagliflozin 100 mg and dapagliflozin 10 mg treatment in adults with T2DM uncontrolled on background metformin therapy. One of the strengths in this study is the application of the COMT model, which has manifested good model validity for established effects of interventions on intermediate endpoints such as glucose, blood pressure and lipid profiles in the Eastern Asian population (Li T. et al., 2018; Wu et al., 2018a).

Several economic studies have reported the cost-effectiveness of SGLT2 inhibitors among adults with T2DM (Tzanetakos et al., 2016; Gourzoulidis et al., 2018; Rahman et al., 2018). Comparing to other oral antidiabetic medicines, such as DPP-4 inhibitor, acarbose and sulfonylurea and dapagliflozin, canagliflozin are likely to be a cost-effective alternative in adults with T2DM. An improvement in HbA1c, an intermediate biomarker, was indicated as a key driver of these cost-effective outcomes. In direct comparison, empagliflozin and canagliflozin may be the more cost-effective in comparison with dapagliflozin (Neslusan et al., 2018), which was coherent with our findings. These findings generally show to be coherent across analytical models, payer perspective, and nations of analysis. However, SGLT2 inhibitors as monotherapy do not appear cost-effective compared with gliclazide or pioglitazone in patients who cannot tolerate the metformin treatment (Johnston et al., 2017).

The univariate sensitivity analyses suggested that the base-case results were relatively robust to adjustments in input variables and assumptions. In almost all univariate sensitivity analyses canagliflozin’s INHB kept above zero QALY gained. We found the most sensitive variables were the cost of canagliflozin and dapagliflozin. When the cost of dapagliflozin reduced near 30%, the canagliflozin’s INHB were below zero QALY gained. The reduction of the cost of canagliflozin enlarge the gaps of INHB between canagliflozin and dapagliflozin strategy. Because the price would be discounted when a drug is covered by the National Reimbursement Drug List of China (Li H. et al., 2018),the economic outcomes needed to be refined when SGLT2 inhibitors is listed. The stability of base case findings was further strengthened by PSA results which produced a relatively high probabilities of cost-effectiveness at the predefined Chinese threshold.

The current analysis have several limitations. The main one was the lack of a clinical trial directly comparing canagliflozin 100 mg strategy and dapagliflozin 10 mg strategy. Due to the absence of a head-to-head study, the results from Bayesian network meta-analysis was adopted, the methods and weaknesses of which have been discussed in the previous report (Zaccardi et al., 2016). Fortunately, the sensitivity analyses showed the uncertainty around the efficacy of canagliflozin and dapagliflozin did not lead to considerably resultant outcomes as would be expected. The current analysis need to be updated when the data of direct comparison become available. Additionally, due to the absence of available long-term health outcomes data of dapagliflozin and canagliflozin as an add-on to metformin on the progression of diabetes-related micro- and macro-vascular consequences, computer modeling techniques was used to predict the risks of diabetes-related complications and mortality as other economic analyses done (Charokopou et al., 2016). Therefore, the long-term comparison of canagliflozin to dapagliflozin has uncertainty, which should be addressed when explaining the findings. However, because our model was validated, the potential uncertainty could maintain to both canagliflozin and dapagliflozin strategies, which might not be considerably different. Finally, the current study did not conduct subgroup analysis. For example, individuals with T2DM and cardiovascular complications might gain more health benefits from the treatment of SGLT2 inhibitors. The current modeling study incorporate cardiovascular disease into the risk equations for predicting the complications, but illustrate the model outcomes reflecting the whole cohort with T2DM. It is unclear if these findings may be extrapolated to diabetic patients without cardiovascular disease which represent a large component of patients.

## Conclusion

This economic analysis found that canagliflozin 100 mg strategy is likely to provide a paucity of health benefits at a relatively less cost compared with dapagliflozin 10 mg strategy for patients with T2DM after the metformin failure in the Chinese health care system. These findings indicate that treatment with canagliflozin 100 mg strategy is a more efficient allocation of limited Chinese health resources for the management of T2DM and may be a factor for patients, physicians, and decision makers in the selection of a reasonable regimen for the treatment of T2DM after the metformin failure.

## Ethics Statement

This economic analysis was based on a literature review and modeling techniques; this study did not require approval by an Institutional Research Ethics Board.

## Author Contributions

BW was involved in the design of the study. BW and XH collected the data. XH and BW developed the model, performed the health economic analysis, and wrote the first draft of the manuscript, which was critically revised by XW and BW.

## Conflict of Interest Statement

The authors declare that the research was conducted in the absence of any commercial or financial relationships that could be construed as a potential conflict of interest.
